# Supplementary material for: Content-rich biological network constructed by mining PubMed abstracts
Source: BMC Bioinformatics. 2004 Oct 8;5:147. doi: 10.1186/1471-2105-5-147 (PMC528731; doi:10.1186/1471-2105-5-147)
Supplement: Additional File 2 — The original results of the above study (non-essential files are deleted to keep the file size under the limit set by BMC bioinformatics). [file 1471-2105-5-147-S2.bz2 › chilibotAdditionalFile2/dip05/527ID8663349E10832/html/CBX5_LBR.html]

 


 **CBX5** and **LBR** 
  
Found 8 abstracts in PubMed, retrieved 05.  
 

 What does Google say? 
 PDF only 
| .edu only 

---

**Interactive relationship** (e.g. stimulation, inhibition, etc)

**Neutral relationship**- These include interactions between nuclear lamins and core histones, Lamina Associated Polypeptide 2 LAP2, and the Barrier to Autointegration Factor BAF and interactions between lamin B receptor  [ **LBR** ]   **LBR**  and the chromodomain protein HP1  [ **CBX5** ] .  Ref: 10651245 Crit Rev Eukaryot Gene Expr, 1999
- Using purified components and in vitro assays, we now demonstrate that HP1  [ **CBX5** ]  forms a quaternary complex with the inner nuclear membrane protein  **LBR**  and a sub set of core histones.  Ref: 11571267 EMBO Rep, 2001
- In this report, we have reconstituted and characterized the HP1  [ **CBX5** ]  KAP 1 interaction using purified proteins and have compared KAP 1 to three other known HP1  [ **CBX5** ]  binding proteins SP100, lamin B receptor  [ **LBR** ]   **LBR** , and the p150 subunit from chromatin assembly factor CAF 1 p150.  Ref: 10938122 Mol Cell Biol, 2000
- The lamins and the lamin binding proteins lamina associated polypeptide LAP 2beta and lamin B receptor  [ **LBR** ]   **LBR**  have been described to bind to DNA or to interact with chromatin via histones, BAF 1, and HP1  [ **CBX5** ]  chromodomain proteins, respectively, and may provide anchorage sites for chromatin fibers at the nuclear periphery.  Ref: 10651242 Crit Rev Eukaryot Gene Expr, 1999

**Non-interactive relationship** (e.g. studied together, co-existance, homology, etc.)

- This complex involves histone H3 H4 oligomers, which mediate binding of  **LBR**  to HP1  [ **CBX5** ]  and cross link these two proteins that do not interact directly with each other.  Ref: 11571267 EMBO Rep, 2001
- Consistent with previous observations, HP1  [ **CBX5** ]  and  **LBR**  binding to core histones is strongly inhibited when H3 H4 are modified by recombinant CREB binding protein, revealing a new mechanism for anchoring domains of under acetylated chromatin to the inner nuclear membrane.  Ref: 11571267 EMBO Rep, 2001
